# Can a Transparent Machine Learning Algorithm Predict Better than Its Black Box Counterparts? A Benchmarking Study Using 110 Data Sets

**DOI:** 10.3390/e26090746

**Published:** 2024-08-31

**Authors:** Ryan A. Peterson, Max McGrath, Joseph E. Cavanaugh

**Affiliations:** 1 Department of Biostatistics & Informatics, Colorado School of Public Health, University of Colorado, Anschutz Medical Campus, 13001 E. 17th Pl, Aurora, CO 80045, USA; 2 Department of Biostatistics, College of Public Health, University of Iowa, 145 N. Riverside Dr., Iowa City, IA 52245, USA; joe-cavanaugh@uiowa.edu

**Keywords:** model selection, feature selection, lasso, explainable machine learning

## Abstract

We developed a novel machine learning (ML) algorithm with the goal of producing transparent models (i.e., understandable by humans) while also flexibly accounting for nonlinearity and interactions. Our method is based on ranked sparsity, and it allows for flexibility and user control in varying the shade of the opacity of black box machine learning methods. The main tenet of ranked sparsity is that an algorithm should be more skeptical of higher-order polynomials and interactions a priori compared to main effects, and hence, the inclusion of these more complex terms should require a higher level of evidence. In this work, we put our new ranked sparsity algorithm (as implemented in the open source R package, sparseR) to the test in a predictive model “bakeoff” (i.e., a benchmarking study of ML algorithms applied “out of the box”, that is, with no special tuning). Algorithms were trained on a large set of simulated and real-world data sets from the Penn Machine Learning Benchmarks database, addressing both regression and binary classification problems. We evaluated the extent to which our human-centered algorithm can attain predictive accuracy that rivals popular black box approaches such as neural networks, random forests, and support vector machines, while also producing more interpretable models. Using out-of-bag error as a meta-outcome, we describe the properties of data sets in which human-centered approaches can perform as well as or better than black box approaches. We found that interpretable approaches predicted optimally or within 5% of the optimal method in most real-world data sets. We provide a more in-depth comparison of the performances of random forests to interpretable methods for several case studies, including exemplars in which algorithms performed similarly, and several cases when interpretable methods underperformed. This work provides a strong rationale for including human-centered transparent algorithms such as ours in predictive modeling applications.

## 1. Introduction

If accurate prediction is the goal, it is a commonly held thought that a model need not be traditionally interpretable. On the contrary, if it helps prediction, the predictors should be allowed to interact freely and associate with the outcome nonlinearly in unfathomable ways. After all, who are we humans to impart our will that a predictive model’s inner workings be understandable?

Since Breiman’s 2001 tale of two cultures [1], the dichotomy between black box prediction and “transparent” statistical models has been the topic of much debate in data science. Black box models are thought to mirror the truly ethereal data-generating mechanisms present in nature; Box’s “all models are wrong” aphorism incarnated into the modeling algorithm itself. These opaque approaches are not traditionally interpretable. Transparent models, on the other hand, we define as traditional statistical models expressed in terms of a linear combination of a maximally parsimonious set of meaningful features. Transparency is reduced as more features are added, especially features that are difficult to interpret (like interactions and polynomials), or those involving complex transformations. Under this definition, transparency is a spectrum where the most transparent model is the “null” model (where new predictions are all set to the expected outcome in the population), followed by single-predictor models, which are often called “unadjusted” models. Our definition resembles that for typical applications of Occam’s Razor in model selection, where the number of parameters in the model translates directly to its simplicity, except that we consider some parameters (interactions, for instance) less transparent than others.

This paper challenges the notion that less transparency actually leads to improvements in predictive accuracy. We have developed an algorithm called the sparsity-ranked lasso (SRL), which prefers transparent statistical models, and we have shown that it outperforms other methods for sifting through derived variables such as polynomials and interactions (when both such relationships truly have signal and more so when they do not) [2]. In this work, we benchmark the performance of the SRL on 110 data sets from the Penn Machine Learning Benchmarking (PMLB) Database [3,4], measuring the extent to which a resulting model’s predictive performance suffers (if it does at all) relative to a set of black box methods. We hypothesize that in many cases, transparent modeling algorithms actually produce better models, and in most cases, they perform comparably to black box alternatives.

Our paper is organized as follows. We first provide a brief overview of the SRL and related methodologies, as well as a description of the black box methods we will use for comparison. We then describe the benefits of transparent approaches over black box approaches from a variety of perspectives, and we set the stage for the experimental comparison of all algorithms applied to 110 data sets from the PMLB, which contain a mix of numeric outcomes (regression tasks) and binary outcomes (classification tasks). In our results section, we describe the data set characteristics and present our model performance both overall and then diving deeper into several illustrative case studies. We conclude with a discussion of our findings in context, describing limitations and suggestions for future work.

## 2. Materials and Methods

### 2.1. Sparsity-Ranked Lasso

Opening Pandora’s box of derived variables, also known as feature engineering, can turn any medium-dimensional problem into an exceptionally high-dimensional one. Even if we restrict these derived variables to include only pairwise interactions or polynomials of existing features, the number of candidate variables grows combinatorically with the number of features *p*. Therefore, we developed a high-dimensional solution to this problem: the sparsity-ranked lasso (SRL).

The SRL was developed as an algorithm based on the Bayesian interpretation of the lasso [5] to favor transparent models (i.e., models with fewer interactions and polynomials). The SRL is based on optimizing the following function with respect to the parameters β, which measure the associations between an outcome *y* and the columns of a covariate matrix *X*:

$$||y-{X\beta ||}^{2}+\lambda \sum_{j=1}^{p}w_{j}\left|\beta_{j}\right|$$

The hyperparameter λ represents the extent of overall shrinkage toward zero, and the nature of the discontinuity in the penalization renders some estimated coefficients exactly zero, inherently deselecting them from the model. The lasso and the SRL are both typically tuned using model selection criteria or cross-validation.

The SRL initially resembles the adaptive lasso [6], using penalty weights $w_{j}$ to increase the penalization (in other words, skepticism) for some columns of *X* and to decrease it for others. In the SRL’s default implementation, the set of supplied covariates (denoted as *A* and henceforth considered “main effects”) becomes supplemented with all of the pairwise interactions (*B*) and second-order polynomials (*C*) as additional columns such that $X=\left[A\;\;B\;\;C\right]$. Without special attention to the relative differences in the size and complexity of these interactions and polynomials in the penalization, the lasso selects too many interactions and polynomials (which renders the model unnecessarily opaque). We have shown that setting $w_{j}=\sqrt{p_{j}}$ for all *j*, where $p_{j}$ represents the size of the set of covariates to which covariate *j* belongs, calibrates the prior information contributed by the collection of interactions to be equal to that of the collection of main effects, naturally inducing skepticism (higher penalties) on interactions without having to tune additional hyperparameters. For polynomial penalization, a slightly modified penalty weight is used based on the cumulative dimension size; see Peterson and Cavanaugh [2] for further details. The SRL is currently implemented in the sparseR R package available on the Comprehensive R Archive Network (CRAN). The SRL can successfully sift through a large, *high-dimensional* set of possible interactions and polynomials while still preferring transparency, which is in contrast to alternative methods that tend to over-select interactions and higher-order polynomials [2,7]. The k and poly arguments to the sparseR function allow the user to tune the maximum order interaction and polynomial, respectively; these values default to k = 1 (all pairwise interactions) and poly = 2 for up-to-second-order polynomials. The log-likelihood loss function replaces the least squares term in the above equation when the outcome is non-Gaussian.

In Peterson and Cavanaugh [2], we used extensive simulation studies to characterize the properties of the SRL, comparing SRL to state-of-the-art competitors for the selection of interactions and polynomials, focusing on predictive accuracy and false discovery rates in the context of generating models that have varying numbers of “true” nonlinear effects (polynomials/interactions). Our results indicated the SRL was superlative in settings where true models were sparse in terms of nonlinear/interacting effects, and especially when no such effects existed. In the high-dimensional setting, where we expect many null relationships, this property is highly advantageous. Furthermore, the strong performance of the SRL was found to hold under varied settings with respect to the correlation structure of the covariates. However, comparing SRL to smoothing splines in lower-dimensional settings, we found the performance to be less favorable when the nonlinear effects could not be well-approximated by polynomials, as well as when the covariates were highly skewed in distribution (though the normalization of skewed covariates partially mitigated the latter issue). In related work, we extended the SRL to time series data, showing via extensive simulations that the SRL could outperform alternatives in settings with complex autoregressive structures or high-dimensional exogenous features [8]. An additional contribution of these simulations was to show that, in addition to finding well-predicting transparent models, the SRL is often computationally quicker than alternatives.

### 2.2. Black Box Algorithms

In this work, we primarily utilize the black box supervised learning algorithms briefly described in this section. Random forest algorithms [9] are an ensemble-based learning method for continuous and categorical endpoints. They operate by constructing many candidate decision trees using bootstrapped and sub-sampled training data, predicting the outcome as the mode of the classes (classification) or mean prediction (regression) of the individual trees. Whereas individual trees (weak learners) may over- or under-fit the training data, using an ensemble improves predictions by averaging multiple decision trees. Support Vector Machines (SVMs) [10] work by finding the hyperplane that best separates observations in the feature space. SVMs are effective in high-dimensional spaces and are particularly useful for cases where the number of features exceeds the number of observations. Extreme Gradient Boosting (XGBoost) [11] is an efficient implementation of the gradient boosting framework. Similarly to random forests, XGboost builds an ensemble of trees, except it does so in a sequential manner, where each tree tries to correct the errors of the previous one. XGBoost also incorporates regularization to prevent overfitting. Neural networks are a set of algorithms inspired by the structure and function of the human brain, designed to recognize patterns [12]. They consist of layers of nodes (neurons) that process input data and pass them through successive layers. Each node assigns weights to its inputs and passes them through an activation function to determine the output. This extremely flexible setup makes neural networks capable of modeling complex, nonlinear relationships. They work particularly well at text, image, and speech recognition. Moreover, a number of different types of architectures have been built for different types of problems, thereby expanding the array of potential applications of the method [13].

### 2.3. Issues with Black Box Algorithms

In classical statistical modeling, the overarching objective is often delineated as either descriptive or predictive. Descriptive modeling focuses on providing a succinct, interpretable characterization of how a set of explanatory variables is jointly associated with the outcome, with the primary inferential goal centered on the estimation and inference of effects (i.e., regression parameters). Predictive modeling focuses on the accurate approximation of new outcomes. A commonly held perspective is that transparency is only an important consideration with descriptive modeling. With large samples, predictive accuracy generally improves as more nuanced and subtle effects are added to the model, leading to a less parsimonious and less interpretable model structure. Black box algorithms are built upon the philosophy that reality is too complex to succinctly encapsulate with a transparent model structure and that optimal prediction is best accomplished by sacrificing interpretability in order to mirror the intricacies and sophistication of reality.

However, in many modeling applications, even if prediction is the primary goal, description is still an important secondary objective. Investigators are generally not only concerned with the quality of the predictions but also with the manner in which they are derived. Without knowing which features are especially important in driving a prediction, or how different variables interact with each other, it becomes difficult to build stakeholder trust in a model. Further, as predictive models are becoming more ubiquitous in society, it is becoming increasingly clear that by hiding biases under the veil of the black box, opaque modeling methods can facilitate unfair systematic discrimination. Outside of biomedical settings, such issues have been described in predictive policing, credit scoring systems, hiring tools, and many more applications [14,15,16,17]. In health settings, such models can perpetuate and exacerbate existing systemic health disparities [18]. In such high-stakes cases when fairness dictates that model-based decisions should be justifiable, opaque modeling methods that worsen disparities are especially problematic; rather than building trust, opaque models tend to erode trust for some while producing excessive trust in others. Transparent models mitigate this issue by making unfair biases on behalf of the model very difficult to hide. Transparency is also important to facilitate the regulation of modern technological innovations, such as autonomous vehicles, smart devices, and large language models. For example, the General Data Protection Regulation (GDPR) provides a legal framework that sets guidelines for the collection and processing of personal information from individuals who live in and outside of the European Union. Adherence to such guidelines may be difficult to achieve by opaque algorithms.

Due to their complexity, black box algorithms can also be difficult to debug or troubleshoot. A related problem is that black box models may degrade over time due to changes in the data distribution (“concept drift”) [19]. Detecting and adapting to the evolution of the data-generating mechanism can be challenging if one is unaware as to which model structures are impacted by the resulting changes.

Additionally, black box algorithms are prone to overfitting and may therefore perform much more effectively in predicting training data than validation data. Moreover, if the features used to build the algorithm are extracted through an automated search as opposed to scientific knowledge, features that are spuriously associated with the outcome may naturally enter the model. Such features may degrade the quality of the prediction if conditions lead to a disconnection in the association. For instance, since the flu season generally coincides with the college basketball season, the number of college basketball games played in a given week during the flu season is typically highly correlated with flu incidence during the same week. However, during atypical flu seasons, such as the 2009 H1N1 pandemic, this association will disappear.

Our philosophy is that a certain degree of complexity is often warranted for high-quality prediction. Yet, a model that is primarily based on meaningful, pronounced features and that only incorporates more nuanced and subtle features if the evidence provided by the data is sufficiently compelling to warrant their inclusion, will often be transparent and interpretable. Moreover, we will subsequently show that such models fit via the SRL or lasso perform as well as or better “out of the box” than a set of popular black box methods that disregard the principle of parsimony and potentially violate Occam’s Razor in a large collection of data sets.

### 2.4. PMLB Processing Steps

PMLB data sets were loaded using the pmlbr R package [20]. Metadata, including predictor types, endpoint types, and feature counts, were extracted from the PMLB GitHub repository (https://github.com/EpistasisLab/pmlb, accessed on 25 June 2024). We restricted analysis of the data sets to those with binary or continuous endpoints (categorical endpoint sets were discarded), with fewer than 10,000 observations, with 50 or fewer predictors, and with fewer than 100,000 total predictor cells (predictor columns times observations). It became evident that simulated data sets based on the Friedman simulation model [21] made up a comparably large fraction of the remaining data sets, and therefore, these were also removed. For categorical predictors, all classes that appeared in less than 10% of observations were combined into a single class. Prior to modeling, all data sets were split into training and test sets, where approximately 20% of observations were set aside in the test set. For each data set, all models were fit and evaluated using the same training and test sets.

### 2.5. Modeling Procedures

As this experiment is intended to be a bakeoff, in that models are compared “out of the box”, algorithms were very minimally tuned.

All random forest, SVM, neural network, and XGBoost models were fit using simple 10-fold cross-validation (CV) and a grid search to tune hyper-parameters. Black box methods were fit with the caret R package [22], which serves as a wrapping package for the following fitting engines: random forests with randomForest [23], SVMs with kernlab [24], feedforward neural networks with nnet [25], and XG-boost with xgboost [26]. The caret package’s defaults were used in all cases; these and other tuning parameters are described in Table S1.

The sparseR package [2] was used to fit SRL and lasso models. By default, the lasso and the SRL use 10-fold CV to search for an optimal value of a single tuning parameter (λ), which controls the overall level of penalization. The SRL fit with sparseR has two noteworthy additional tuning parameters that can be modified manually: k, which refers to the number of order interactions to consider, and poly, which refers to the maximum order polynomial to consider. The default value for k is 1, which searches among all pairwise interactions. The default value for poly is 2, which searches for up-to order 2 polynomials and thereby allows for limited nonlinearity of features. The sparseR package uses the ncvreg package as a backend fitting engine [27]. Further modifications are available; see ?sparseR for more detailed documentation.

For numeric outcomes, we tuned all algorithms with CV-based root mean squared error (RMSE), and we also computed the CV-based R-squared (its traditional formulation using the sum of squared errors) for evaluation. The RMSE and R-squared measure the aggregate distance between an observation’s model-based prediction and its true value. The RMSE measures this distance in the same unit as the outcome of interest, while the R-squared does so in a unitless fashion, where a value of 0 indicates that the model performs identically to predicting the mean value for all observations (i.e., no predictive value of the model), and a value of 1 means perfect prediction. Similarly, we computed test set-based R-squared and RMSE values for each combination of algorithm and PMLB data set for evaluation. Binary endpoints were tuned using CV-based deviance for the lasso and the SRL (sparseR’s default) and CV-based accuracy for methods trained with caret (its default). While both binomial deviance and accuracy are meant to assess the quality of a model’s predictions, the former also considers prediction “confidence” in its computation; a highly confident, yet incorrect, prediction is penalized worse than a less certain, though still incorrect, prediction. Binary endpoints were evaluated using the area under the receiver operating characteristic curve (AUC) for each model’s predictions on the test set. The AUC quantifies the overall ability of the model to classify observations. Models are simpler to compare with the AUC than the accuracy when classes are imbalanced; a value of 1 indicates perfect prediction, whereas a value of 0.5 indicates that a model is no better than randomly guessing the outcome based on the overall proportion of observations in each class. In some cases, the out-of-bag R-squared estimate was negative; in those instances, the R-squared was set to zero prior to subsequent modeling.

### 2.6. Meta-Modeling for Inference

To perform inferences on the differences in average performance across modeling algorithms, we fit linear mixed models to the outcomes of CV-based R-squared, out-of-sample R-squared, and AUC values. In these models, each data set received a random intercept to account for data set-specific differences in the signal-to-noise ratio. We included fixed effects for the modeling algorithm, with our SRL serving as the baseline for inference. Comparisons between the SRL and competitors were assessed using the lmerTest package, which uses Satterthwaite’s approximated degrees of freedom for coefficient hypothesis tests [28].

## 3. Results

### 3.1. Data Set Characteristics

**Figure 1:**
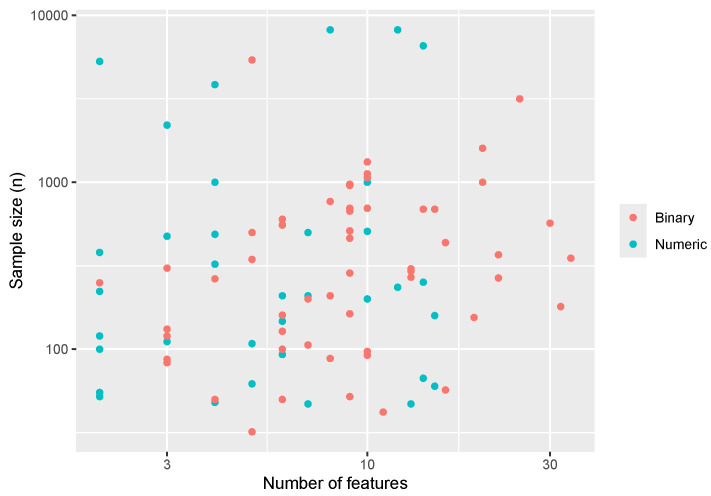
Overview of data set sizes in the Penn Machine Learning Benchmarks database.

Descriptive statistics for our sampled PMLB data sets are presented in Table 1 for the overall sample and stratified by endpoint type. The size of the data sets (sample size vs number of features) is visualized in Figure 1, showing a fairly uniform distribution along our studied range of features and sample sizes for both categorical and continuous endpoint types. On average, data sets had five categorical features (standard deviation (SD): 7), and five continuous features (SD: 6).

### 3.2. Overall Model Performance

Descriptive results for model performances are shown in Table 2. For continuous endpoints, the lasso and SRL had the best-performing model for test data in 12.8% and 17.9% of the data sets (totaling 30.7%), and the SRL was within 5% out-of-sample predictive accuracy of the best performing model in nearly two thirds of data sets. For binary endpoints, the lasso and SRL performed best in 22.7% and 34.8% of the data sets (totaling 57.5%), and the SRL was within 5% of the best model in 78.8% of the data sets. The lasso and SRL were generally faster than the black box methods.

**Figure 2:**
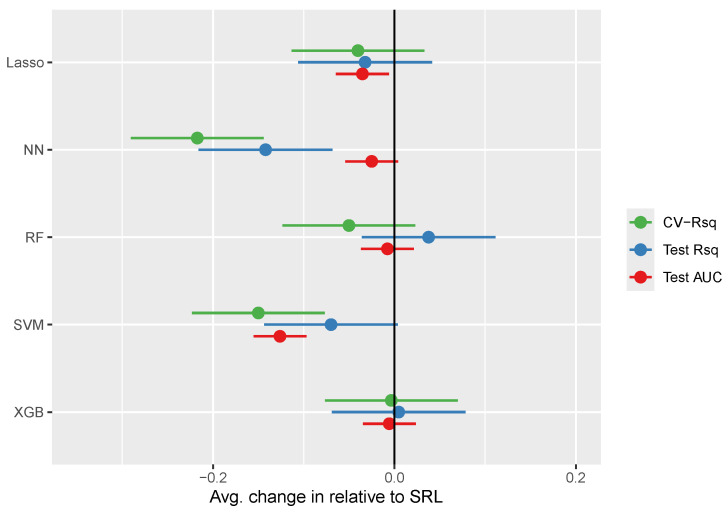
Linear mixed model results contrasting the expected change in predictive accuracy compared to SRL, controlling for data set-specific prediction difficulty. CV: cross-validation. AUC: area under the receiver operator curve.

Inferential results comparing models in terms of CV-based R-squared, out-of-sample R-squared, and out-of-sample AUC values are displayed in Table 3 and summarized in Figure 2. The SRL generally performed slightly better than the lasso, though this difference was only significant for binary endpoints, where the SRL had test set mean AUCs that were 3.5 percentage points higher (95% CI: 1–6; *p* = 0.018). Similarly, the SRL generally performed significantly better than neural networks and SVMs across most outcome metrics. Random forests and XG-boosting performed generally similar to SRL, with all performance comparisons being insignificant.

**Figure 3:**
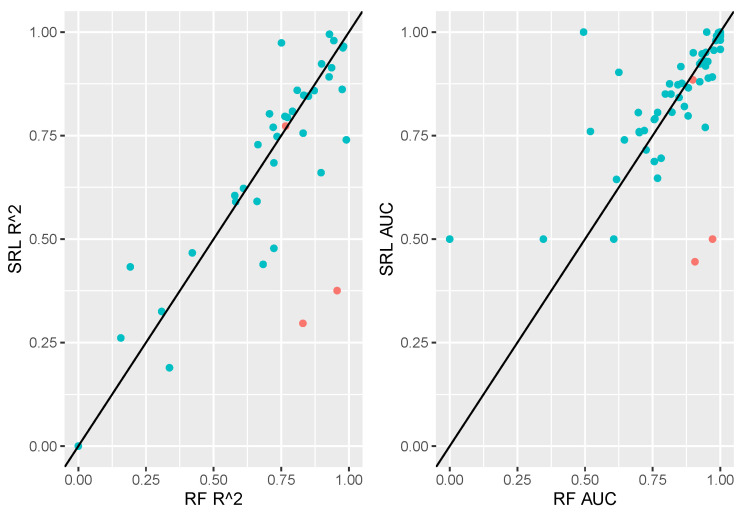
Comparing the predictive performance of random forests to that of SRL on held-out test sets. Each point represents a data set.

Figure 3 displays a comparison of random forests to the SRL in terms of out-of-sample performance for all data sets. Here we note that random forests and SRL performed similarly on the majority of data sets. There are a handful of cases in which random forests highly outperformed the SRL. A subset of data sets denoted in Figure 3 as red points will be investigated in the next section as illustrative case studies.

### 3.3. Case Studies

Here, we present six case studies, starting with two exemplars of the pattern evident in Figure 3, where the SRL and random forests models performed similarly, and concluding with four outliers, where the SRL seemed to be underperforming relative to random forests.

#### 3.3.1. Exemplars

**Figure 4:**
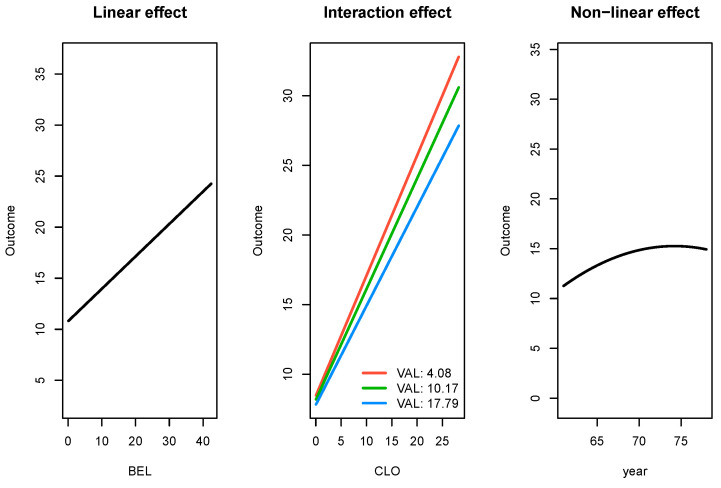
For the 503 wind data set, SRL discovered significant and interpretable linear relationships (**left**), interaction effects (**center**), and nonlinear relationships (**right**).

The first case study is the 503_wind data set, for which the PMLB goal is to predict daily average wind speed at a weather station in Malin Head, Ireland, based on the date and the wind speeds recorded by 11 weather stations in the Republic of Ireland in the years 1961–1978 [29]. This data set has 6574 observations, and it is further summarized in Table S2. We found that the SRL outperformed all other methods in terms of test R-squared and test RMSE, with a notably faster run time than the random forests, SVMs, and, to a lesser extent, neural network methods. Results for the 503_wind data set are provided in Table 4. In addition to the SRL being the best performer, it also produced parameter estimates that are interpretable. In Figure 4, we present the effects for three types of significant relationships found by the SRL in the 503_wind data: linear, linear with an interaction effect, and a nonlinear effect.

**Figure 5:**
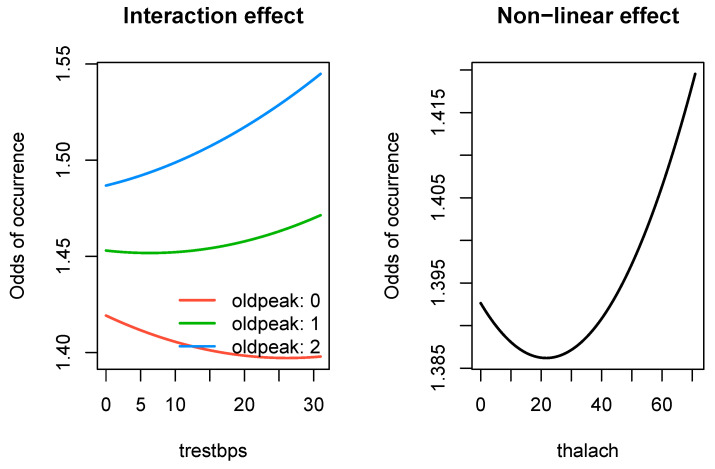
For the hungarian data set, SRL discovered significant and interpretable interaction relationships (**left**), as well as a meaningful quadratic relationship (**right**).

The second case study is the hungarian data set, which consists of a subset of patients undergoing catheterization at the Hungarian Institute of Cardiology in Budapest between 1983 and 1987 [30]. The PMLB prediction goal is to predict the presence of heart disease based on a set of 14 variables (summarized in Table S3). The SRL was the fourth best performing model in terms of the AUC; however, the performance of the top four models was extremely close, with each having an AUC value within 0.032 of one another. Results for the hungarian data set are provided in Table 5. While the SRL did not outperform random forests for this data set, it did provide interpretable parameter estimates relative to random forests for only a marginal reduction in performance. In Figure 5, we present the effect for two types of significant nonlinear relationships found by the SRL in the hungarian data: an interaction effect and a quadratic effect.

#### 3.3.2. SRL Underperforming RFs

In this section, we delve more deeply into examples where the SRL appears to be performing worse than alternative methods (case studies highlighted in Figure 3 to the right of the 45-degree line); these data sets are named analcatdata_apnea1, analcatdata_apnea2, analcatdata_boxing1, and parity5+5. Descriptive statistics for all of the variables included in these data sets are shown in Tables S4–S6. The apnea-related data sets are set up as regression tasks (numeric outcomes) by the PMLB, and they are originally described in Steltner et al. [31]. The other data sets, analcatdata_boxing1 and parity5+5, are binary classification tasks, but we were unable to trace them to their original sources.

For the sleep apnea data sets analcatdata_apnea1 and analcatdata_apnea2, the SRL, lasso, and SVMs performed considerably worse in terms of test and cross-validated $R^{2}$ values compared to random forests and XGboost (Table 6).

**Figure 6:**
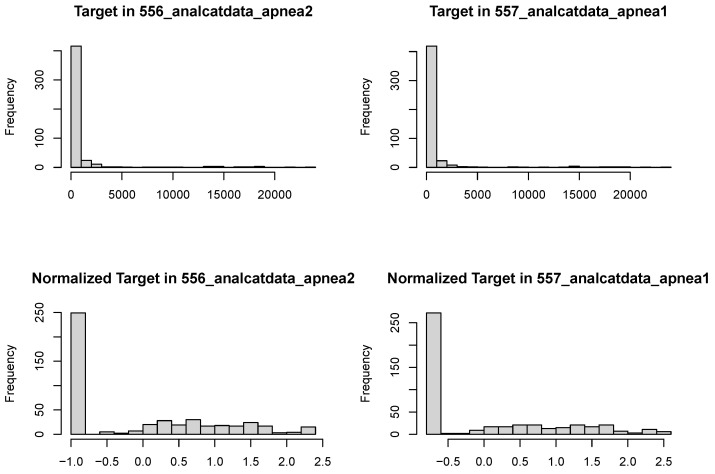
Distributions of target variables for sleep apnea data sets (**top**: raw; **bottom**: normalized).

Examining the target outcomes for these data sets (Figure 6), we see that both outcomes are highly skewed with a point mass at zero, rebutting even normalization methods [32,33]. Given these distributions, it makes sense for the models to be fit better by more robust methods. While SRL (and lasso) algorithms could be introduced that adequately capture zero inflation and right skew, that is beyond the scope of this paper.

Upon further inspection, we noticed that the sparseR package by default removes interactions or other terms with near-zero variance via the recipes package [22,34], which in this case removed all of the candidate interaction features from the model prior to the supervised part of the algorithm. By adding the argument filter = "zv", only zero-variance variables are removed, and therefore, any interactions with variance are retained. The code for applying this solution and its results are shown in the Supplementary Materials. Once this was implemented for the analcatdata_apnea2 data set, the SRL achieved a CV-based R-squared value of 0.91, and a compact model (within one standard error of the RMSE of the best model) achieved a CV-based R-squared value of 0.88. Coefficients from the latter model and their marginal false discovery rates [35] can be viewed in the Supplementary Materials as well. Briefly, we can interpret the model as follows: observations with Automatic ∈{0,3} or those where Scorer_1 ∈{0,3} were associated with higher values of the target variable. If Automatic = 0 and Scorer_1 = 0, there was a multiplicative modest increase in the target, but when both variables were equal to three, the target jumped up to the extremely high tail of the distribution, increasing by over 13,000 on average. These results are practically identical for the analcatdata_apnea1 data set.

**Figure 7:**
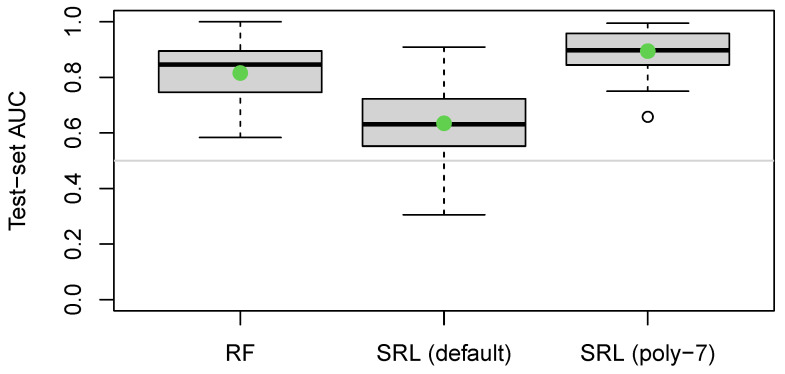
Distribution of test set area under the receiver operator curve (AUC) for random forests (RFs, **left**), SRL (default, **middle**), and SRL with up to seven-order polynomials selected (**right**) for 50 different train/test splits for the analcatdata_boxing1 data set.

For the analcatdata_boxing1 and parity5+5 data sets, the results are summarized in Table 7. The analcatdata_boxing1 data set contains 120 observations and only three variables: Official (binary), Round (integer from 1–12), and the target. Due to the small sample size, we repeated the train–test split many times and noticed that, while there was substantial variability in the test AUC, the SRL still performed worse than the random forests method. We suspected that the difference may have been due to a complex nonlinear relationship between Round and the target. By default, sparseR only looks for pairwise interactions, main effects, and second-order polynomials, but it is readily extendible higher-order polynomials (while still preferring lower-order terms; see Peterson and Cavanaugh [2] and Peterson [7]). Here, setting poly = 7 allows for up to seventh-order polynomials to be considered. The results for all three models are shown in Figure 7, in which we confirm that this additional flexibility with SRL yields notably better predictions.

The parity5+5 data set consists of 1124 observations, 10 binary predictors, and a single binary target variable. It seems to us to be designed to showcase a scenario where transparent modeling methods are set up for failure. The target variable for this data set uses the nonlinear parity function based on a random subset of size five of the features. In this case, we used the built-in variable importance metrics for the random forests to discover that the subset of “important” features were the second, third, fourth, sixth, and eighth features. We could then confirm the importance of these variables by summing these binary features and recognizing that the outcome was always 1 when this subset sum was even and always 0 otherwise. Finally, we note that adding this summation as a candidate feature to the SRL and adding polynomial terms to sparseR did improve the model fit considerably, but as this modification requires a hybrid approach (i.e., it blends information from random forests and SRL), it does not provide a fair comparison of our method to black box methods, and we do not describe these results.

## 4. Discussion

We are not the first to suggest that transparent modeling methods perform comparably to black box methods; Christodoulou et al. [36] found that when aggregating across biomedical data sets from 71 real studies, logistic regression performed, on average, exactly the same as black box alternatives.

Data sets are growing increasingly large and diverse, and the subset of data set examples we explored in the PMLB, while larger than any previous study comparing such methods, is limited in generalizability to data sets with similar outcomes, numbers of features, signal-to-noise ratios, and variable distributions. In particular, we cannot generalize these findings to especially high-dimensional data sets (p>50) or massive data sets (n>10,000 or np>100,000), as these were not included in our analysis. This comparison and extension would be welcome in future work, as black box models are said to be data-hungry, performing best in these massive data settings [37]. However, this extension would require the improved scalability of various methods (including the SRL) as currently implemented. Another limitation to our study is the fact that the PMLB database has sparse metadata available for its data sets, and we were unable to trace many of the data sets back to their original sources.

Given the currently available methods and software, the SRL and lasso are less readily applied to quantitative outcomes whose distributions involve a high degree of non-normality. In such cases, random forests and other robust algorithms may outperform our transparent ones. However, robust transparent modeling algorithms might also be considered in such settings such as robust regression or quantile regression. In our example, we found that a simple tweak to the defaults in the SRL yielded a model on par with black box modeling, but we suspect this fix may only apply to data sets with large signal-to-noise ratios; often a predictor capable of delineating different outcome modes is not available.

We did not investigate the implementation of stacking or other ensemble-based approaches [38,39]. Under our definition of transparency, such approaches are not transparent. Therefore, if a transparent model fits the data best, it will improve the performance of black box ensembles, but at a high cost of reduced interpretability. Still, in practice it is advisable to fit such an ensemble and compare its performance to transparent methods alone. One can compare the relative weight of transparent methods against black box alternatives to map the data set-specific tradeoff between predictive accuracy and transparency and then make decisions regarding whether an observed improvement in performance (if it exists) is worth the opacity, as well as its potential issues regarding trust, fairness, stability, etc.

In our paper, we have considered the SRL and the lasso as two techniques that can be used to produce transparent models. However, numerous algorithms and methods are available that are designed to achieve the same objective. As stated in the introduction, we define transparent models as traditional statistical models expressed in terms of a linear combination of a maximally parsimonious set of meaningful features. Such models are often developed by initially formulating a general parametric model that includes all potential candidate variables, along with any derived variables (e.g., transformed variables, polynomials, interactions, etc.) that may seem plausible a priori. A variable selection algorithm is then applied to reduce the complexity of the model and arrive at an interpretable final model that better adheres to the principle of Occam’s Razor.

Two common statistical approaches to variable selection are based on optimizing a penalized likelihood measure and optimizing an information criterion. The SRL and the lasso are both penalized likelihood methods. Other such methods include the elastic net, the adaptive lasso, the fused lasso, and the relaxed lasso. Information criterion approaches involve using a penalized measure of model fit, such as the Akaike information criterion or the Bayesian information criterion, in conjunction with a search algorithm that evaluates all or some of the fitted models in the candidate collection using the criterion values. Best subsets selection is an exact algorithm based on an exhaustive search and yields a final model that is guaranteed to optimize the criterion. Heuristic algorithms exchange exactness for computational efficiency and/or simplicity, and they may not necessarily identify the globally optimal model, but they will hopefully yield a model that is nearly optimal (i.e., has a criterion value close to the global minimum/maximum). Classical stagewise algorithms, such as forward selection and backward elimination, are examples of heuristic algorithms.

In addition to techniques based on penalized likelihood and information criteria, many other algorithms and techniques that facilitate transparent modeling have been proposed, developed, and studied. For instance, decision trees, including classification trees and regression trees, can often yield a transparent model through a sequence of well-defined, hierarchical variable splits. Another important paradigm is the Logical Analysis of Data (LAD) [40,41,42], which is a methodological framework designed to extract or discover knowledge from data in a logical form. The LAD combines concepts from optimization, combinatorics, and Boolean functions for data analysis.

Similarly, due to the bakeoff nature of this experiment, we only compared algorithms using default values chosen by existing software packages, namely those used by caret for the black box approaches. An important question is whether the algorithms we use for comparison can be considered to represent the state of the art. We chose the most popular packages openly available in R via the CRAN website for fitting neural networks (nnet), random forests (randomForest), support vector machines (kernlab), and XG-boosting (xgboost); at the time of writing, these packages each had (by far) more cumulative downloads from CRAN than other packages within each model class. These packages are undoubtedly popular due to their accessibility, generalizability to new problems, and historical precedence, making them good candidates for our experiment. However, more recently developed algorithms in each model class, including those not yet openly available via R or CRAN, are likely to outperform existing popular packages. Therefore, we do not claim that our models will necessarily outperform or compete well to the state of the art; rather, we expect that our method will compete admirably when compared to the most popular modeling alternatives. Future comparisons of more recently developed, state-of-the-art algorithms, as well as models using more involved tuning strategies, would be welcome.

In this paper, we focused on comparisons between algorithms “in the wild” (i.e., on real data sets), where the true data-generating mechanisms are naturally unknown. This focus builds substantively atop our previous work, and it showcases concretely how transparent methods deserve more attention and popularity. We plan to conduct a similar type of analysis using our time series SRL extension on a large, diverse collection of time series. Still, there is ample room for future research in silica to investigate the SRL’s performance under varied scenarios. Specifically, the robustness of the SRL to extreme outliers, noise intensity, the presence of gaps in the distributions of covariates, and highly irregular covariate correlation structures may cause issues that deserve additional attention in future work. Nevertheless, we have shown herein that the SRL can compete admirably despite the presence of such issues in real data sets, at least for the purposes of prediction.

In conclusion, our transparent algorithms sometimes predict better than black box counterparts and most of the time perform comparably. We encourage modelers to always at least consider a transparent modeling approach, even in applications where prediction is the main objective.

## Figures and Tables

**Table 1:** Means (standard deviations) of data set characteristics. Class imbalance refers to a measure of class distribution of the target (outcome) variable, with a value approaching 0 indicating perfectly balanced target classes and a value approaching 1 indicating extreme class imbalance, where nearly all instances belong to one class. N refers to the number of data sets under study.

Characteristic	Overall, N = 110	Binary, N = 69	Numeric, N = 41
Sample size	856.21 (1619.0)	611.93 (795.8)	1267.32 (2406.2)
Number of features	10.15 (7.0)	12.07 (7.6)	6.93 (4.4)
Number of numeric features	5.14 (6.0)	4.10 (6.5)	6.88 (4.5)
Number of categorical features	5.02 (7.0)	7.97 (7.4)	0.05 (0.3)
Class imbalance	0.08 (0.1)	0.11 (0.2)	0.04 (0.1)

**Table 2:** Performance across all data sets. SRL: sparsity-ranked lasso. NN: neural networks. RFs: random forests. SVMs: support vector machines. XGB: extreme gradient boosting.

	SRL	Lasso	NN	RFs	SVMs	XGB
**Continuous**						
CV Rsq; mean (SD)	69.4 (23)	65.4 (22)	47.7 (29)	64.4 (26)	54.4 (28)	69.1 (20)
Test Rsq; mean (SD)	68.2 (25)	65 (25)	54 (31)	72 (24)	61.2 (26)	68.7 (26)
Best performance (%)	17.9	12.8	20.5	35.9	15.4	10.3
Within 5% of best (%)	61.5	35.9	35.9	59.0	35.9	46.2
Run time (s); mean (SD)	3.9 (3)	2.6 (2)	8.1 (9)	16.4 (17)	10.6 (13)	15.6 (5)
**Binary**						
Test AUC; mean (SD)	85.9 (15)	82.4 (17)	83.4 (16)	85.1 (18)	73.3 (18)	85.3 (16)
Best performance (%)	34.8	22.7	27.3	37.9	6.1	39.4
Within 5% of best (%)	78.8	65.2	56.1	69.7	18.2	71.2
Run time (s); mean (SD)	11.6 (11)	7.2 (8)	12.7 (10)	13.9 (14)	8.3 (8)	14.9 (3)

**Table 3:** Linear mixed (meta) models. Estimates refer to the expected change in the prediction outcome relative to SRL controlling for data set-specific prediction difficulty. The Int. (intercept) term refers to the expected value for the listed measure for the SRL. SRL: sparsity-ranked lasso. NNs: neural networks. RFs: random forests. SVMs: support vector machines. XGB: extreme gradient boosting. ***p*** refers to the mixed model-based ***p***-value testing the hypothesis that each coefficient is equal to zero.

Term	CV Rsq		Test Rsq		AUC	
	**Estimate (CI)**	***p***	**Estimate (CI)**	***p***	**Estimate (CI)**	***p***
Int.	69.4 (62, 77)	<0.001	68.2 (60, 77)	<0.001	85.9 (82, 90)	<0.001
Lasso	−4 (−11, 3)	0.28	−3.2 (−11, 4)	0.39	−3.5 (−6, −1)	0.018
NNs	−21.7 (−29, −14)	<0.001	−14.2 (−22, −7)	<0.001	−2.5 (−5, 0)	0.092
RFs	−5 (−12, 2)	0.18	3.8 (−4, 11)	0.32	−0.8 (−4, 2)	0.60
SVMs	−15 (−22, −8)	<0.001	−7 (−14, 0)	0.063	−12.6 (−16, −10)	<0.001
XGB	−0.3 (−8, 7)	0.93	0.5 (−7, 8)	0.90	−0.6 (−3, 2)	0.70

**Table 4:** Comparison of performance for the 503_wind set. SRL: sparsity-ranked lasso. NNs: neural networks. RFs: random forests. SVMs: support vector machines. XGB: extreme gradient boosting.

Model	Test R-Squared	Test RMSE	Run Time (s)
SRL	0.773	3.12	12.8
Lasso	0.741	3.34	8.4
RFs	0.766	3.17	48.7
SVMs	0.744	3.32	34.8
NNs	0.667	3.78	17.3
XGB	0.770	3.14	4.1

**Table 5:** Comparison of performance for the hungarian data set. SRL: sparsity-ranked lasso. NNs: neural networks. RFs: random forests. SVMs: support vector machines. XGB: extreme gradient boosting.

Model	AUC	Runtime (s)
SRL	0.885	5.8
Lasso	0.894	2.5
RFs	0.899	7.9
SVMs	0.821	9.6
NNs	0.917	10.5
XGB	0.811	18.1

**Table 6:** Comparison of performance for the sleep apnea data sets. SRL: sparsity-ranked lasso. NNs: neural networks. RFs: random forests. SVMs: support vector machines. XGB: extreme gradient boosting. s: seconds.

Model	R-Squared (CV)	R-Squared (Test)	Run Time (s)
**556_analcatdata_apnea2**			
SRL	0.247	0.376	1.8
Lasso	0.243	0.385	1.3
RFs	0.760	0.956	20.4
SVMs	0.111	0.021	11.6
NNs	0.292	0.719	6.2
XGB	0.684	0.930	17.9
**557_analcatdata_apnea1**			
SRL	0.276	0.296	1.7
Lasso	0.297	0.299	1.1
RFs	0.810	0.830	17.6
SVMs	0.082	0.039	8.9
NNs	0.635	0.823	6.3
XGB	0.859	0.820	19.1

**Table 7:** Comparison of performance for binary outcome data sets where SRL underperformed. SRL: sparsity-ranked lasso, NNs: neural networks. RFs: random forests. SVMs: support vector machines. XGB: extreme gradient boosting. AUC: area under the receiver operator curve. s: seconds.

Model	AUC	Runtime (s)
**analcatdata_boxing1**		
SRL	0.445	2.6
Lasso	0.758	1.5
RFs	0.906	1.6
SVMs	0.594	3.5
NNs	0.727	2.3
XGB	0.898	14.4
**parity5+5**		
SRL	0.500	18.0
Lasso	0.500	6.8
RFs	0.971	1.2
SVMs	0.500	4.1
NNs	0.990	31.1
XGB	0.443	12.5

## Data Availability

All code and data resulting from this manuscript are publicly available at https://github.com/petersonR/sparser-bakeoff-public. The source data are available at https://github.com/EpistasisLab/pmlb.

## Supplementary Materials

The following supporting information can be downloaded at https://www.mdpi.com/article/10.3390/e26090746/s1. Table S1: Model specification in R; Table S2: Descriptive statistics including median and interquartile range for the 503_wind data set; Table S3: Descriptive statistics including medians and interquartile ranges for numeric variables and counts/percentages for categorical variables for the Hungarian data set, stratified by the classification target, which takes values of 0 or 1; Table S4: Descriptive statistics including medians and interquartile ranges for numeric variables and counts/percentages for categorical variables in the sleep apnea data sets; Table S5: Descriptive statistics including counts and percentages for the parity5+5 data set, stratified by the classification target which takes values of 0 or 1; Table S6: Descriptive statistics including including medians and interquartile ranges for the numeric variable and counts/percentages for the categorical variable for the analcatdata_boxing1 data set, stratified by the classification target which takes values of 0 or 1.

## Abbreviations

The following abbreviations are used in this manuscript:

SRL	Sparsity-ranked lasso
PMLB	Penn machine learning benchmarks (database)
RF	Random forest
SVMs	Support vector machines
NNs	Neural networks
XG-Boost (XGB)	Extreme gradient boosting
AUC	Area under the receiver operator curve
RMSE	Root mean squared error
CV	Cross-validation
SD	Standard deviation
LAD	Logical analysis of data
