# Supplementary material for: Can a Transparent Machine Learning Algorithm Predict Better than Its Black Box Counterparts? A Benchmarking Study Using 110 Data Sets
Source: Entropy (Basel). 2024 Aug 31;26(9):746. doi: 10.3390/e26090746 (PMC11431724; doi:10.3390/e26090746)
Supplement: Supplementary file 1 [file entropy-26-00746-s001.zip › entropy-3103777-supplementary.pdf]

Appendix to: Can a Transparent Machine Learning Algorithm Predict Better than Its Black-Box Counterparts? A Benchmarking Study using 110 Datasets

Supplemental Tables

Table S1: Model specification in R.

Algorithm	Package	Tuning Parameters
Random Forest	`randomForest`*	$p \in \{1, 2, ..., \}$; maximum depth $\in \{2, 3, 4\}$
Neural Network	`nnet`*	number of hidden units $\in \{1, 2, ..., 5\}$; decay $\in \{0, 10^{-1}, ..., 10^{-4}\}$
XGBoost	`xgboost`*	maximum depth $\in \{1, 2, 3\}$; maximum iterations $\in \{50, 100, 150\}$; $\eta \in \{0.3, 0.4\}$; subsample ratio $\in \{0.5, 0.75, 1\}$
Support Vector Machines	`kernlab`*	C (cost)=1
Lasso	`sparseR`	$\lambda \in (0, \infty)$
Sparsity-Ranked Lasso	`sparseR`	$\lambda \in (0, \infty)$; `k` = 1; `poly` = 2

Note:

*`caret` used as wrapping package which specifies defaults listed.

Note: In the tables that follow describing specific Penn Machine Learning Benchmarks (PMLB) data sets, we adhere to the capitalization and naming conventions of each specific data set (variable names are not modified in tables or captions). Additional descriptions as well as source citations for these data sets (if available) are included in Section 3.3 of the main manuscript. Please note that these tables present our pre-processed versions of the data sets (with the processing steps outlined in Section 2.4 of the main manuscript), so they may not align perfectly with the data sets as downloaded from the PMLB.

Table S2: Descriptive statistics including median and interquartile range for the 503_wind data set.

**Characteristic**	**N = 6,574**
year	69.5 (65.0, 74.0)
month	7.0 (4.0, 10.0)
day	16 (8, 23)
RPT	11.7 (8.1, 15.9)
VAL	10.2 (6.7, 14.0)
ROS	10.9 (8.0, 14.7)
KIL	5.8 (3.6, 8.4)
SHA	10.0 (6.8, 13.5)
BIR	6.8 (4.0, 9.7)
DUB	9.2 (6.0, 13.0)
CLA	8.1 (5.1, 11.4)
MUL	8.2 (5.4, 11.2)
CLO	8.3 (5.3, 11.6)
BEL	12.5 (8.7, 16.9)
target	15 (11, 20)

[1] Median (IQR)

Table S3: Descriptive statistics including medians and interquartile ranges for numeric variables and counts/percentages for categorical variables for the Hungarian data set, stratified by the classification target, which takes values of 0 or 1.

**Characteristic**	**0, N = 188**	**1, N = 106**
age	48 (41, 54)	50 (46, 54)
sex		
0	69 (37%)	12 (11%)
1	119 (63%)	94 (89%)
cp		
1	7 (3.7%)	4 (3.8%)
2	98 (52%)	8 (7.5%)
3	43 (23%)	11 (10%)
4	40 (21%)	83 (78%)
trestbps	14 (9, 19)	17 (9, 22)
chol	68 (37, 110)	84 (51, 112)
fbs		
0	175 (93%)	91 (86%)
Other	13 (6.9%)	15 (14%)
restecg		
0	150 (80%)	85 (80%)
1	33 (18%)	19 (18%)
Other	5 (2.7%)	2 (1.9%)
thalach	34 (23, 46)	29 (15, 41)
exang		
0	168 (89%)	36 (34%)
1	19 (10%)	70 (66%)
2	1 (0.5%)	0 (0%)
oldpeak	0.00 (0.00, 0.00)	1.00 (0.00, 2.00)
slope		
1	19 (10%)	72 (68%)
3	157 (84%)	33 (31%)
Other	12 (6.4%)	1 (0.9%)
ca		
0	2 (1.1%)	1 (0.9%)
1	186 (99%)	105 (99%)
thal		
3	173 (92%)	93 (88%)
Other	15 (8.0%)	13 (12%)

[1] Median (IQR); n (%)

Table S4: Descriptive statistics including medians and interquartile ranges for numeric variables and counts/percentages for categorical variables in the sleep apnea data sets.

	Apnea 2	Apnea 1
Variable	**N = 475**	**N = 475**
Automatic		
0	95 (20%)	
1	95 (20%)	
2	95 (20%)	
3	95 (20%)	
4	95 (20%)	
Scorer_1		
0	95 (20%)	95 (20%)
1	95 (20%)	95 (20%)
2	95 (20%)	95 (20%)
3	95 (20%)	95 (20%)
4	95 (20%)	95 (20%)
Subject	10.0 (5.0, 15.0)	10.0 (5.0, 15.0)
target	0 (0, 113)	0 (0, 75)
Scorer_2		
0		95 (20%)
1		95 (20%)
2		95 (20%)
3		95 (20%)
4		95 (20%)

[1] n (%); Median (IQR)

The table above contains summary information on all of the variables in the data sets called `analcatdata_apnea1` and `analcatdata_apnea2`. The variables called `Scorer_1`, `Subject`, and `target` were shared across these two data sets. `Subject` refers to subject-specific identifiers but is treated as a numeric variable by default by all methods. This may seem like a questionable decision. However, we decided adhering to the default was the most appropriate way to handle the variable due to the nature of setting up the experiment as a bakeoff; we could not have any special rules for variables based on their name being something similar to "Subject".

Table S5: Descriptive statistics including counts and percentages for the parity5+5 data set, stratified by the classification target which takes values of 0 or 1.

**Characteristic**	**0, N = 557**	**1, N = 567**
Bit.1		
0	285 (51%)	289 (51%)
1	272 (49%)	278 (49%)
Bit.2		
0	283 (51%)	289 (51%)
1	274 (49%)	278 (49%)
Bit.3		
0	276 (50%)	284 (50%)
1	281 (50%)	283 (50%)
Bit.4		
0	278 (50%)	283 (50%)
1	279 (50%)	284 (50%)
Bit.5		
0	278 (50%)	290 (51%)
1	279 (50%)	277 (49%)
Bit.6		
0	280 (50%)	286 (50%)
1	277 (50%)	281 (50%)
Bit.7		
0	279 (50%)	283 (50%)
1	278 (50%)	284 (50%)
Bit.8		
0	280 (50%)	286 (50%)
1	277 (50%)	281 (50%)
Bit.9		
0	280 (50%)	278 (49%)
1	277 (50%)	289 (51%)
Bit.10		
0	284 (51%)	293 (52%)
1	273 (49%)	274 (48%)

[1] n (%)

Table S6: Descriptive statistics including including medians and interquartile ranges for the numeric variable and counts/percentages for the categorical variable for the analcatdata_boxing1 data set, stratified by the classification target which takes values of 0 or 1.

**Characteristic**	**0, N = 42**	**1, N = 78**
Official		
0	23 (55%)	61 (78%)
1	19 (45%)	17 (22%)
Round	9 (4, 10)	6 (2, 8)

[1] n (%); Median (IQR)

Example Code and Output from sparseR

The code below uses the analcatdata_apnea2 data set from the PMLB and models the data with the SRL setting filter = "zv", as described in our main manuscript. The reader is encouraged to confirm this solution works similarly with the analcatdata_apnea1 data set.

```
set.seed(1233)
(srl <- sparseR(target ~ ., data = analcatdata_apnea2, filter = "zv"))
```

```
##
## Model summary @ min CV:
## -----------------------------------------------------
##   lasso-penalized linear regression with n=475, p=47
##   (At lambda=3.8098):
##     Nonzero coefficients: 17
##     Cross-validation error (deviance): 892294.26
##     R-squared: 0.91
##     Signal-to-noise ratio: 9.85
##     Scale estimate (sigma): 944.613
##
##   SR information:
##              Vartype Total Selected Saturation Penalty
##          Main effect    11        6      0.545    3.32
##  Order 1 interaction    35       10      0.286    5.92
##   Order 2 polynomial     1        1      1.000    3.46
##
##
## Model summary @ CV1se:
## -----------------------------------------------------
##   lasso-penalized linear regression with n=475, p=47
##   (At lambda=47.1308):
##     Nonzero coefficients: 6
##     Cross-validation error (deviance): 1135592.07
##     R-squared: 0.88
##     Signal-to-noise ratio: 7.53
##     Scale estimate (sigma): 1065.642
##
##   SR information:
##              Vartype Total Selected Saturation Penalty
##          Main effect    11        4     0.3636    3.32
##  Order 1 interaction    35        2     0.0571    5.92
##   Order 2 polynomial     1        0     0.0000    3.46
```

```
summary(srl, at = "cv1se")
```

```
## lasso-penalized linear regression with n=475, p=47
## At lambda=47.1308:
## -------------------------------------------------
##   Nonzero coefficients         :   6
##   Expected nonzero coefficients:   0.02
##   Average mfdr (6 features)    :   0.003
##
```

```
##                             Estimate      z      mfdr Selected
## Automatic_X3:Scorer_1_X3 13510.346 60.547   < 1e-04        *
## Automatic_X0:Scorer_1_X0     6.923  5.797   < 1e-04        *
## Scorer_1_X0                100.114  4.062 0.0010881        *
## Automatic_X3                75.760  3.862 0.0021474        *
## Automatic_X0                60.563  3.734 0.0035268        *
## Scorer_1_X3                 35.016  3.524 0.0090114        *
```
